# Transarterial Radioembolization for the Treatment of Advanced Hepatocellular Carcinoma Invading the Right Atrium

**DOI:** 10.1007/s00270-020-02605-3

**Published:** 2020-08-05

**Authors:** Raphaël Girardet, Sarah Boughdad, Antonia Digklia, Catherine Beigelman, Marie Meyer, Niklaus Schaefer, Mathilde Vermersch, Arnaud Hocquelet, Georgia Tsoumakidou, Alban Denys, Rafael Duran

**Affiliations:** 1 Department of Radiology and Interventional Radiology, Lausanne University Hospital, University of Lausanne, Rue du Bugnon 46, 1011 Lausanne, Switzerland; 2 Department of Nuclear Medicine and Molecular Imaging, Lausanne University Hospital, University of Lausanne, Lausanne, Switzerland; 3 Department of Medical Oncology, Lausanne University Hospital, University of Lausanne, Lausanne, Switzerland

**Keywords:** HCC, Radioembolization, Immune pneumonitis

## Abstract

Hepatocellular carcinoma (HCC) has the tendency to invade the portal and/or hepatic venous system. The invasion of the right atrium is uncommonly observed and constitutes a treatment challenge. We report the case of a patient with HCC invading the right atrium treated with ^90^Yttrium-transarterial radioembolization (^90^Y-TARE). Following the treatment, organizing pneumonia secondary to nivolumab occurred, raising the question of an interaction between ^90^Y-TARE and nivolumab.

**Electronic supplementary material:**

The online version of this article (10.1007/s00270-020-02605-3) contains supplementary material, which is available to authorized users.

## Introduction

Data about the treatment of hepatocellular carcinoma (HCC) invading the right atrium is scarce. A few reports investigated different strategies (surgery, radiofrequency ablation, transarterial chemoembolization (TACE), radiation therapy or chemotherapy) [1–4]. However, there is no consensus. The prognosis is dismal with or without treatment (median survival: 1–4 months) [5].

We report the case of a patient with HCC invading the right atrium who benefited from ^90^Yttrium-transarterial radioembolization (^90^Y-TARE). We discuss the rationale and risks of ^90^Y-TARE in this subgroup of patients.

## Case

Patient’s specific consent was obtained for this report. A 71-year-old male with alcoholic cirrhosis (Child–Pugh A5) presented in the emergency department with abdominal pain and hemodynamic instability. CT scan showed multifocal HCC involving segments II, VII, VIII, with spontaneous rupture of a 9-cm tumor (segment VII), and hemoperitoneum. The patient was successfully treated with superselective Gelfoam embolization, with complete tumor necrosis on follow-up imaging. Several locoregional treatments of the remaining lesions were performed over an 18-month period; conventional TACE (cTACE) of left liver and segments VII/VIII, and radiofrequency ablation of tumor in segment VIII. The α-fetoprotein dropped from 182 to 15.3 ng/ml.

**Fig. 1:**
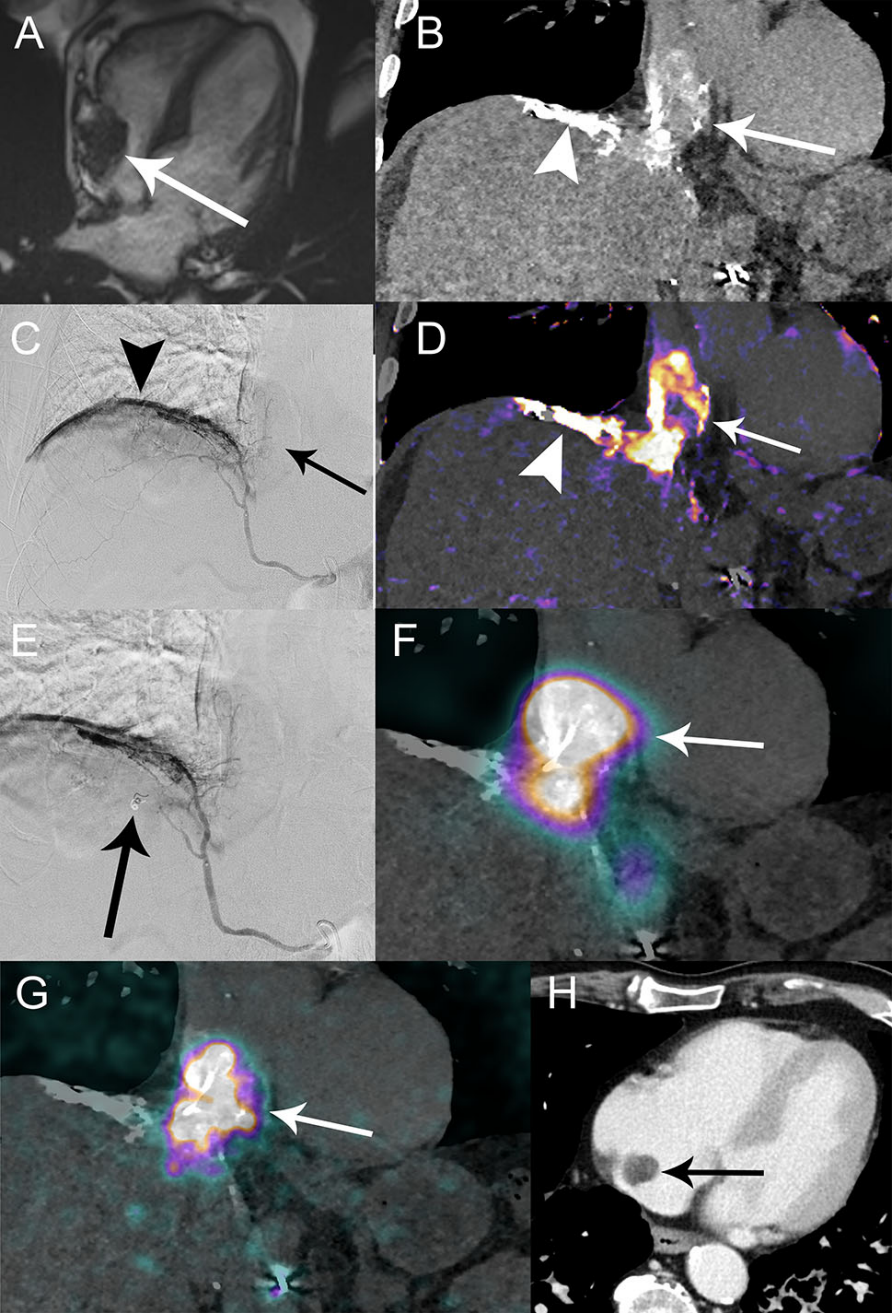
**A** Pre-treatment cardiac MRI: right atrium tumor extension (5.1 × 3 cm) (arrow). **B**–**D** Pre-treatment angio-CT (**B**), digital subtraction angiography (DSA) (**C**) and subtraction CT (**D**) obtained with the injection of iodinated contrast through a microcatheter (Progreat 2.7 Fr, Terumo) positioned in the right inferior phrenic artery (rIPA) shows contrast uptake of the atrial tumor (arrows) and diaphragm (arrowheads). **E** DSA: microcoil (Tornado 0.018 inch/3–2 mm, Cook) embolization (arrow) of the posterior branch of rIPA to protect as much diaphragm as possible and prioritize the blood flow to the tumor. Pre-treatment angio-CT fused with **F** ^99m^Tc-MAA-SPECT and **G** post-treatment ^90^Y-PET demonstrate marked uptake by the atrial tumor (arrow). **H** Contrast-enhanced axial CT image at 5 months post-^90^Y-TARE demonstrates marked decrease of the atrial tumor extension (black arrow) measuring 3.2 × 2.2 cm

Five months after the last treatment, follow-up imaging showed a tumor thrombus invading the right hepatic vein, inferior vena cava (IVC) and right atrium, arising from a small tumor infiltrating segment VII. The patient was asymptomatic. Cardiac MRI demonstrated a 4 × 2.6 cm right atrial mass (Supplementary movie 1/Fig. 1A). No anticoagulation was administered as the thrombus had tumor features on imaging. Sorafenib was deemed inappropriate due to bleeding/thromboembolic risks and patient’s comorbidities, and nivolumab was administered for 2 months (240 mg every 2 weeks). A month later, progression of right atrium mass (5.1 × 3 cm; tumor growth rate (TGR) of 11%/month over the previous 3 months) prompted emergency treatment. ^90^Y-TARE was decided at the tumor board in an attempt to stop tumor progression at the atrial level. Surgery was contraindicated due to multifocal disease with venous invasion, and TACE was considered perilous because of the risk of atrial tumor rupture.

**Fig. 2:**
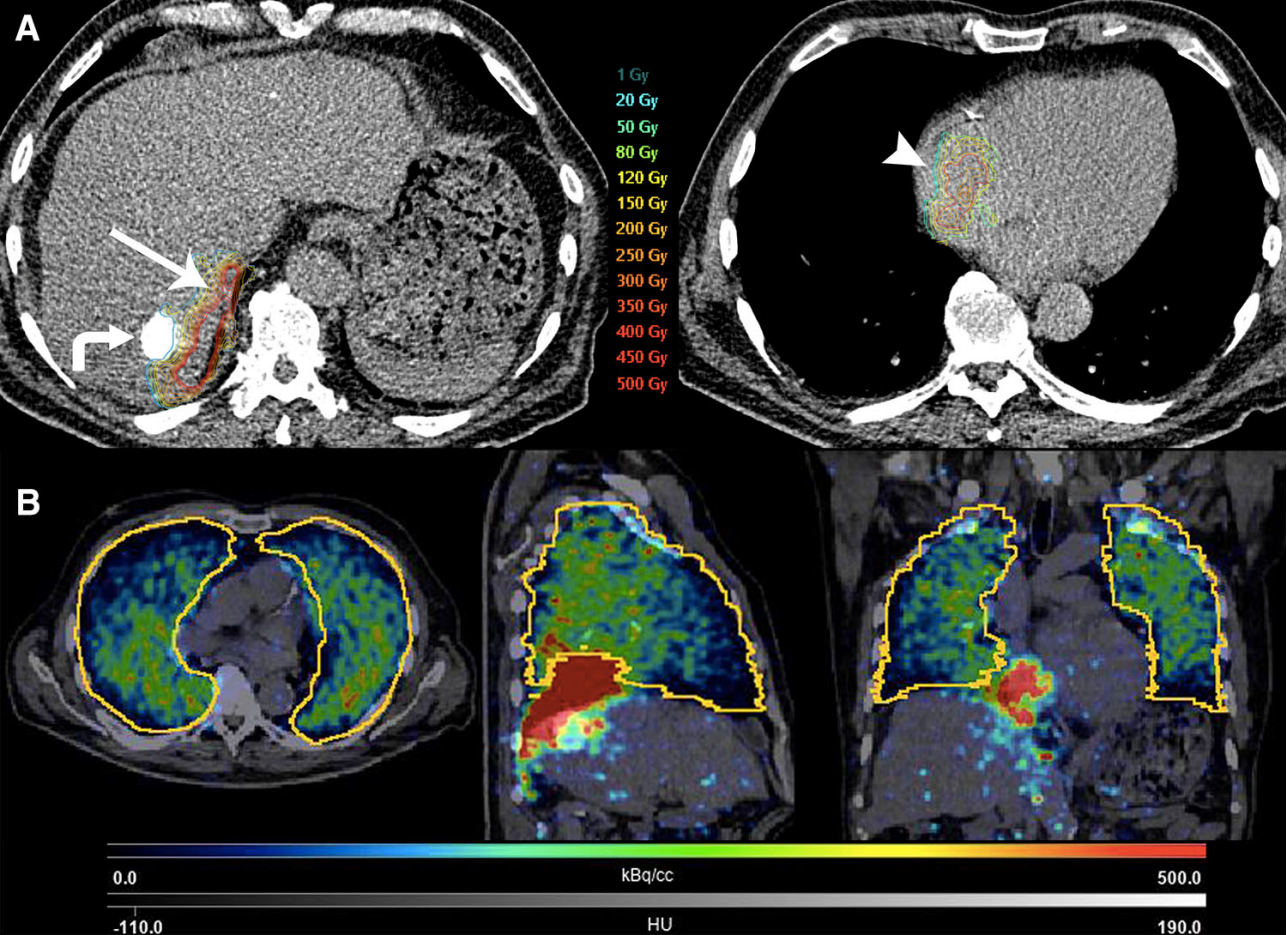
**A** Post-treatment ^90^Y-PET-CT dose distribution (Simplicit^90^Y/BTG) to the atrial tumor (arrowhead) and adjacent diaphragm (arrow). Previously treated HCC lesion filled with Lipiodol (curved arrow). **B** Post-treatment ^90^Y-PET-CT multiplanar dose distribution (PMOD Technologies) in the lungs with a gradient, predominant in the posterior parts

Pre-treatment angiography and CT demonstrated exclusive tumor arterial supply via the right inferior phrenic artery (rIPA) (Fig. 1B–D). Microcoil embolization was performed in the posterior branch of rIPA to protect the diaphragm and prioritize the blood flow to the tumor (Fig. 1E). ^99m^Technetium-macroaggregated albumin (^99m^Tc-MAA) scintigraphy showed a 30% lung shunt fraction (LSF) with an estimated calculated dose of 216.7 Gy to the tumor and 19.4 Gy to the lungs, for an administered activity of 1.3 GBq. A week later, ^90^Y-TARE was performed with injection of 1.3 GBq SIR-Spheres (Sirtex Medical) through the rIPA. Post-administration, ^90^Y-PET/CT showed an absorbed dose of 194.2 Gy to the tumor, 240.7 Gy to the adjacent diaphragm, 17.3 Gy to the lung and 22.3 Gy to non-tumoral liver (Fig. 2).

At 2-month follow-up MRI, the atrial tumor had decreased in size (4 × 2.3 cm; TGR:-15%/month). New bilobar small HCC nodules and a retroperitoneal metastatic lymphadenopathy appeared. At 5-month CT, residual atrial tumor had markedly decreased (3.2 × 2.2 cm; TGR: − 14%/month since ^90^Y-TARE). Hepatic lesions and retroperitoneal lymphadenopathy progressed. The patient refused further therapies and was still alive at 10 months post-^90^Y-TARE.

Interestingly, our patient developed organizing pneumonia secondary to nivolumab during follow-up, raising questions about potential interactions between ^90^Y-TARE and immune checkpoint inhibitor therapy (Supplementary Material 1 and Supplementary Figures 3 & 4).

## Discussion

The main finding of our report is that ^90^Y-TARE of HCC invading the right atrium was feasible and effective in a rapidly growing tumor.

HCC invading the right atrium is a treatment challenge, and efficacy of available treatments is yet to be proven. Conventional TACE is the most frequently utilized catheter-based treatment for HCC invading the right atrium with dismal outcomes [4, 5]. The largest series reported the outcomes of 26 patients with invasion of the IVC including 5 patients with coexisting tumor extension into the right atrium who were treated with cTACE. The median overall survival was 4.2 months [5]. HCC rupture following TACE is a rare but feared complication. Risk factors include male sex, large tumor size, subcapsular location and exophytic outgrowth [6]. These factors need to be considered in light of predictors of spontaneous rupture, such as arterial hypertension, cirrhosis, tumor size > 5 cm, vascular thrombus and extrahepatic invasion [7]. Taken together, the absence of substantial demonstrated efficacy of TACE, and TACE-induced risk of tumor edema/rupture in this fast-growing mass (per se at risk of cardiopulmonary embolism/collapse) prompted us to favor ^90^Y-TARE.

In our patient, the ^99m^Tc-MAA-scintigraphy showed a 30% LSF. Published upper limit for resin ^90^Y-microspheres shunt fraction is 20%, and treatment is not recommended beyond this limit [8]. To avoid radiation-induced lung injury (RILI), a mean lung absorbed dose up to 30 Gy for one radioembolization and a cumulative mean lung absorbed dose up to 50 Gy for repeated radioembolizations are empirically recommended for resin and glass ^90^Y-microspheres [9]. For resin ^90^Y-microspheres, this 30 Gy lung dose maximum is used for establishing the > 20% LSF contraindication threshold, should the entire 3 GBq-vial be administered. For glass ^90^Y-microspheres, no LSF contraindication threshold is specified. A recent study analyzed 103 HCC patients treated with glass ^90^Y-micropsheres with LSF > 15%. The median LSF was 24.4%. The median lung dose per session and cumulative lung dose were 22.9 and 29.5 Gy. Twenty patients (19%) developed non-specific pulmonary complaints in the first year, none attributable to ^90^Y-TARE, and no RILI was observed [10].

Collectively, these data show that both LSF percentage and the absolute dose delivered to the lungs are important factors. The LSF percentage per se as a unique variable should not prevent selected patients from being treated with ^90^Y-TARE, and the absolute dose delivered to the lungs should be the main limiting factor in treating those patients. In our patient, the balance between benefits of ^90^Y-TARE and risk of RILI favored treatment, especially since ^99m^Tc-MAA-based dose calculation estimated a lung absorbed dose of 19.4 Gy. Thus, no prophylactic measures were taken to decrease the hepatopulmonary shunting (such as bland embolization, [11]), and we aimed for an atrial tumor dose of > 200 Gy, as a dose reduction would imply decreased efficacy and potential futility of ^90^Y-TARE itself.

In conclusion, ^90^Y-TARE is a palliative treatment option for patients with advanced HCC extending up to the right atrium. An increased LSF per se should not prevent ^90^Y-TARE from being performed and is not as important as the lungs absolute absorbed dose. Further research is needed to investigate the safety of ^90^Y-TARE performed shortly after immune checkpoint inhibitor therapy.

## Electronic supplementary material

Below is the link to the electronic supplementary material.

Supplementary material 1 (DOCX 26 kb)

Supplementary Figure 3 Timeline of treatments and events (TIFF 768 kb)

Supplementary Figure 4 A) Chest CT at 2.5 months post-^90^Y-TARE: patchy ground glass opacities in a peribronchovascular and subpleural location (arrowheads), relatively sparing the posterior part of the lungs. B)-C) Fusion of chest CT at 2.5 months post-treatment with ^90^Y-PET-CT in axial (B) and sagittal (C), shows a mismatch of lung lesions (predominant in the anterior parts) and ^90^Y-microspheres distribution (predominant in the posterior parts). D) Chest CT at 3 months post-treatment shows progression to alveolar consolidation of subpleural and peribronchovascular localization (arrowheads), with dilated bronchi and distortion of fissures. E)-F) Fusion of chest CT at 3 months post-treatment with ^90^Y-PET-CT in axial (E) and sagittal (F) shows a mismatch of lung lesions (predominant in the anterior parts) and ^90^Y-microspheres distribution (predominant in the posterior parts) (TIFF 4804 kb)

Supplementary video 1 Breath-hold, retrospective ECG-gating cardiac cine using steady-state free precession (SSFP, also known as true-FISP) (1.5T MAGNETOM Sola, Siemens, Erlangen, Germany) (MP4 46696 kb)
