# Supplementary material for: Transarterial Radioembolization for the Treatment of Advanced Hepatocellular Carcinoma Invading the Right Atrium
Source: Cardiovasc Intervent Radiol. 2020 Aug 5;43(11):1712–5. doi: 10.1007/s00270-020-02605-3 (PMC7591430; doi:10.1007/s00270-020-02605-3)
Supplement: Supplementary file 1 — Supplementary material 1 (DOCX 26 kb) [file 270_2020_2605_MOESM3_ESM.docx]

**Supplementary Material:**

**Organizing pneumonia secondary to nivolumab**

At 2.5 months post-^90^Y-TARE (**Supplementary Figure 3**), a follow-up CT done at an outside private facility showed new bilateral patchy upper lobe predominant ill–defined ground glass opacities, sometimes mixed with alveolar consolidation, in a mostly peribronchovascular and subpleural distribution, with slightly enlarged bronchi and fissural retraction **(Supplementary Figure 4A)**. At this point, the patient had not complained about any symptoms such as dyspnea. Two weeks later, the patient came to the emergency room for the advent of progressive shortness of breath and cough. On clinical examination, there was marked dyspnea with severe hypoxemia (partial pressure of oxygen (PaO2) of 63 mmHg in arterial blood), prompting hospitalization of the patient. When asking the patient, dyspnea had been retrospectively slowly appearing for 1.5 months. On repeated chest CT, a worsening was observed in extent with predominant areas of alveolar consolidation in a peribronchovascular and subpleural distribution with migratory lesions, along with architectural distortion (traction bronchiectasis and fissure distortion) **(Supplementary Figure 4D)**. Bronchoscopy with bronchoalveolar lavage was performed, revealing 42.5% of macrophages, 14.5% of neutrophils, 2.5% of eosinophils and 36% of lymphocytes, compatible with organizing pneumonia. No putative pathogens were found.

Radiation-induced lung injury (RILI) seemed unlikely, as the reported delay to develop radiation-induced fibrosis is 6–24 months (1). Indeed, two phases of RILI can be distinguished: 1) Pneumonitis which occurs 1-3 months after treatment and presents with progressive dyspnea, non-productive cough (60%) and fever (< 10%). Chest radiographs will show diffuse haziness in irradiated lung areas. On CT scans, radiation-induced pneumonitis presents as confluent ill-defined patchy opacities and ground-glass nodularity in a symmetric pattern, with relative peripheral/hilar sparing. 2) Fibrosis can occur 6–24 months after treatment. It can be asymptomatic or manifest as a restrictive pattern and pulmonary hypertension. On CT scans, it presents with focal fibrosis signs, such as traction bronchiectasis, architectural destruction and honeycombing (2, 3). Moreover, and importantly, there was a relative sparing of the posterior part of the lung on chest CT, whereas post-^90^Y-TARE ^90^Y-PET/CT showed predominant ^90^Y-microspheres deposition in the posterior, declining, parts of the lungs (**Supplementary** **Figure 4**). A final diagnosis of secondary organizing pneumonia due to toxicity following nivolumab therapy was agreed on, and treatment with IV methylprednisolone for 5 days (followed by *per os* prednisone 60mg/day) together with empiric co-amoxicillin (1g 3x/day for 5 days) and prophylactic Bactrim (160 mg trimethoprim/800 mg sulfamethoxazole, 3x/week) was started. Within 2 weeks, the respiratory symptoms slowly improved with disappearance of cough and residual dyspnea, treated with oxygen (1.5l/min at rest and 2l/min at effort). Radiologic findings improved on subsequent imaging, with decrease in extent of alveolar consolidations but persistence of architectural distortion.

With the advent of immune checkpoint inhibitors, and new molecules entering the market every year, adverse events related to those treatments are becoming more prevalent. Lung adverse events following anti-programmed cell death 1 therapies are infrequent (~1-3% of patients) but potentially life-threatening. They may occur at any time, but most commonly several months after treatment is initiated, as in our patient (4 months post-nivolumab) (4, 5). In our patient, findings from the ^90^Y-PET-CT demonstrating an actual absorbed lung dose of 17.3 Gy and a mismatch between visible lung lesions and ^90^Y-microspheres deposition (higher in declining portions – **Supplementary Figure 4**) plead against a direct lung lesion by ^90^Y-TARE itself. However, as secondary organizing pneumonia developed shortly after ^90^Y-TARE, it raises the question of an interaction between ^90^Y-TARE and recent nivolumab treatment, in particular if ^90^Y-TARE could have triggered this immune pneumonitis (6). This question remains unanswered and will need further investigations. Caution must be taken in patients undergoing ^90^Y-TARE shortly after immune checkpoint inhibitor therapy, and further research is needed.

**References**

1. Graves PR, Siddiqui F, Anscher MS, Movsas B. Radiation Pulmonary Toxicity: From Mechanisms to Management. Seminars in Radiation Oncology. 2010;20(3):201-7.

2. Graves PR, Siddiqui F, Anscher MS, Movsas B. Radiation pulmonary toxicity: from mechanisms to management. Seminars in radiation oncology. 2010;20(3):201-7.

3. Leung TW, Lau WY, Ho SK, Ward SC, Chow JH, Chan MS, et al. Radiation pneumonitis after selective internal radiation treatment with intraarterial 90yttrium-microspheres for inoperable hepatic tumors. Int J Radiat Oncol Biol Phys. 1995;33(4):919-24.

4. Topalian SL, Hodi FS, Brahmer JR, Gettinger SN, Smith DC, McDermott DF, et al. Safety, Activity, and Immune Correlates of Anti–PD-1 Antibody in Cancer. New England Journal of Medicine. 2012;366(26):2443-54.

5. Friedman CF, Proverbs-Singh TA, Postow MA. Treatment of the Immune-Related Adverse Effects of Immune Checkpoint Inhibitors: A Review. JAMA Oncol. 2016;2(10):1346-53.

6. Schoenfeld JD, Nishino M, Severgnini M, Manos M, Mak RH, Hodi FS. Pneumonitis resulting from radiation and immune checkpoint blockade illustrates characteristic clinical, radiologic and circulating biomarker features. J Immunother Cancer. 2019;7(1):112.
