# Supplementary figures and images for: Transarterial Radioembolization for the Treatment of Advanced Hepatocellular Carcinoma Invading the Right Atrium
Source: Cardiovasc Intervent Radiol. 2020 Aug 5;43(11):1712–5. doi: 10.1007/s00270-020-02605-3 (PMC7591430; doi:10.1007/s00270-020-02605-3)

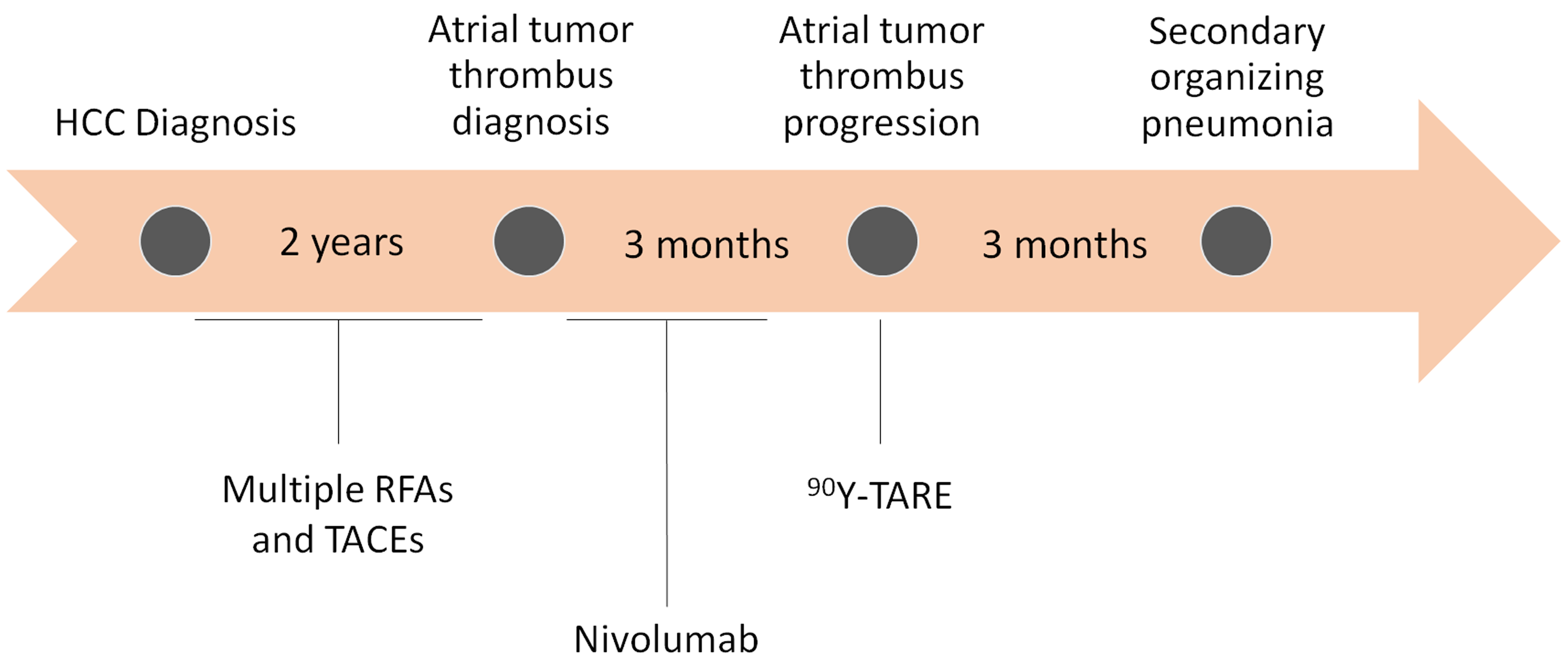

Supplement: Supplementary file 2 — Timeline of treatments and events (TIFF 768 kb) [file 270_2020_2605_MOESM1_ESM.tif]

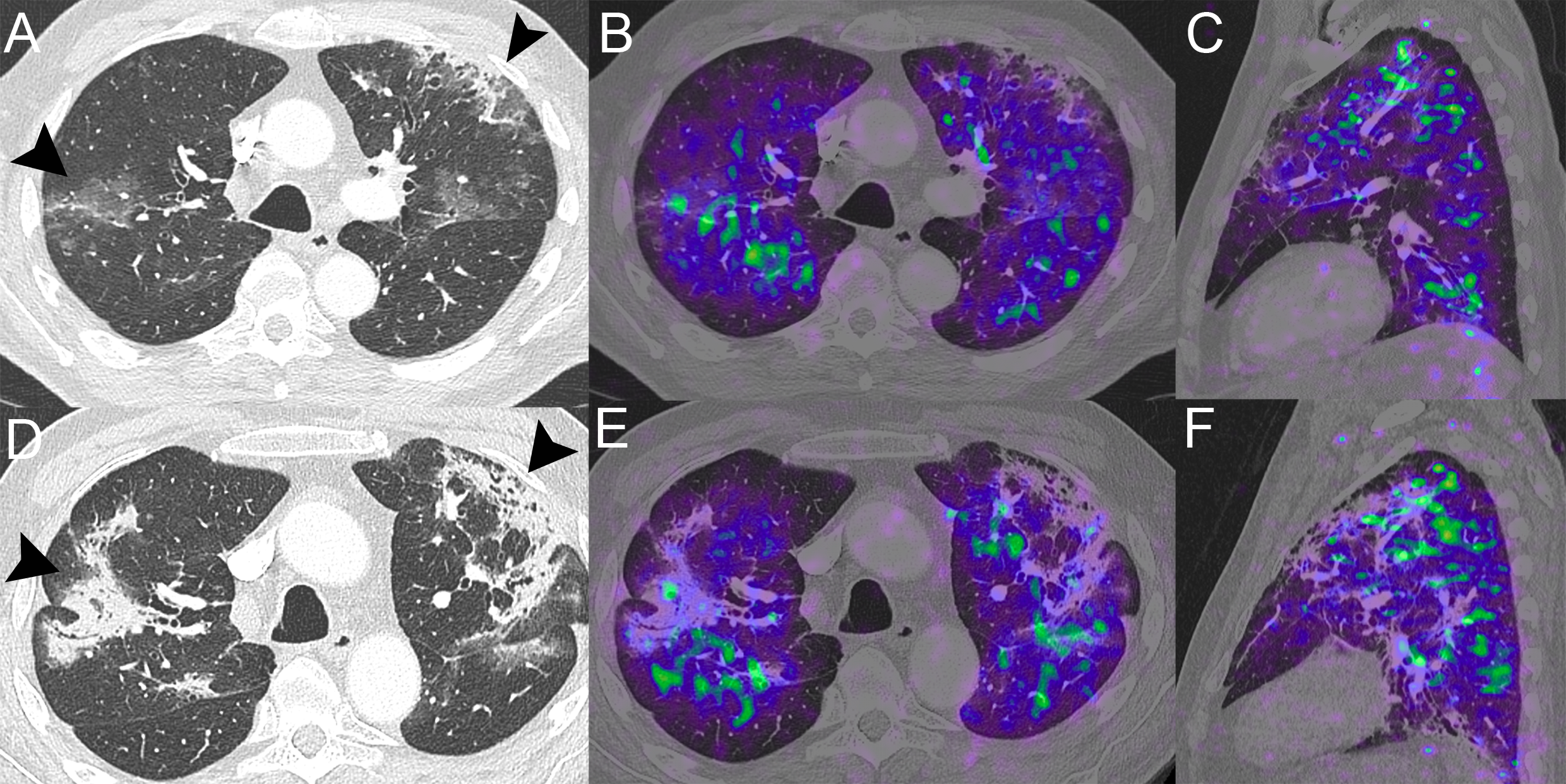

Supplement: Supplementary file 3 — A) Chest CT at 2.5 months post-90Y-TARE: patchy ground glass opacities in a peribronchovascular and subpleural location (arrowheads), relatively sparing the posterior part of the lungs. B)-C) Fusion of chest CT at 2.5 months post-treatment with 90Y-PET-CT in axial (B) and sagittal (C), shows a mismatch of lung lesions (predominant in the anterior parts) and 90Y-microspheres distribution (predominant in the posterior parts). D) Chest CT at 3 months post-treatment shows progression to alveolar consolidation of subpleural and peribronchovascular localization (arrowheads), with dilated bronchi and distortion of fissures. E)-F) Fusion of chest CT at 3 months post-treatment with 90Y-PET-CT in axial (E) and sagittal (F) shows a mismatch of lung lesions (predominant in the anterior parts) and 90Y-microspheres distribution (predominant in the posterior parts) (TIFF 4804 kb) [file 270_2020_2605_MOESM2_ESM.tif]
